# Enhanced Key Node Identification in Complex Networks Based on Fractal Dimension and Entropy-Driven Spring Model

**DOI:** 10.3390/e27090911

**Published:** 2025-08-28

**Authors:** Zhaoliang Zhou, Xiaoli Huang, Zhaoyan Li, Wenbo Jiang

**Affiliations:** 1 School of Electrical Engineering and Electronic Information, Xihua University, Chengdu 610039, China; zhouzhaoliang@stu.xhu.edu.cn (Z.Z.); jiangwenbo@mail.xhu.edu.cn (W.J.); 2 Department of Physics, Fribourg University, 1700 Fribourg, Switzerland; 3 School of Computer and Software Engineering, Xihua University, Chengdu 610039, China

**Keywords:** complex networks, identify key node, information entropy, fractal technology, spring model, node influence range, attenuation factor

## Abstract

How to identify the key nodes in a complex network is a major challenge. In this paper, we propose a Second-Order Neighborhood Entropy Fuzzy Local Dimension Spring Model (SNEFLD-SM). SNEFLD-SM model combines a variety of centrality methods based on spring model, such as second-order neighborhood centrality, betweenness centrality, and fractal dimension, to evaluate the importance of nodes. Fractal technology can effectively boost the framework’s proficiency in understanding network self-similarity and hierarchical structure in multi-scale complex networks. It overcomes the limitation of the traditional centrality method which only focuses on local or global information. The method introduces information entropy and node influence range; information entropy can effectively capture the local and global features of the network. The node influence rangecan increase the node importance distinction and reduce the calculation cost. Meanwhile, an attenuation factor is introduced to suppress the “rich-club” phenomenon. Tests on six networks show that SNEFLD-SM has higher accuracy in critical node detection than traditional methods. Furthermore, the application of information entropy further strengthens the model’s capability to recognize key nodes.

## 1. Introduction

Complex networks have a very powerful modeling ability in the real world, which can abstract research targets into nodes and transform relationships between targets into links. For example, email networks [1] represent email communication between users, collaborative networks [2] represent cooperative relationships between authors, and protein networks [3] abstract functional relationships between proteins into links to help people gain insight into biochemical reactions. Understanding the architecture of intricate networks and their information flow mechanisms is crucial. Complex networks can facilitate scientific research, such as controlling public opinion and the spread of deceptive news [4], using influential nodes in social networks to spread commodity information [5] and suppress the spread of viruses [6].

With the development of key node identification methods, scholars have established diverse techniques for evaluating node centrality, such as degree centrality (DC) [7], semi-local centrality (SLC), betweenness centrality (BC) [8], eigenvector centrality (EC) [7], closeness centrality (CC), and PageRank algorithm [9], they all have their own emphases. For example, degree centrality focuses on the information of neighbor nodes. Betweenness and closeness centrality measures emphasize the paths between nodes. PageRank approach represents a fundamental technique for node recognition that relies on feature vectors. Each of these algorithms has its own advantages and limitations. Since the dimension of complex networks can reveal the topology and dynamic structure of networks, Silva et al. proposed local dimension (LD) based on power-law distribution characteristics in complex networks. Pu et al. improved it to identify critical nodes [10]. Integrating concepts from information theory and LD, the research community has put forward local information dimension (LID) [11] and local fuzzy information centrality (LFIC) [12]. Utilizing fuzzy set theory enables precise characterization of inter-node relationships based on their topological distance, providing enhanced accuracy in network description. In view of this, Wen and colleagues introduced a fuzzy local dimension metric for identifying influence nodes, which is suitable for most complex networks [13]. Moreover, information entropy serves as a metric for evaluating node significance. Qiao et al. proposed entropy centrality and described the association between node pairs as a potential for communication activity [14]. In order to obtain more informative and broader information, a novel methodology was developed by Li et al., founded on entropy and mutual information centrality (EMI) [15]. Zareie et al. proposed a new method based on information theory, which can detect the propagation ability of nodes according to their topological information [16]. Although many scientists have conducted a lot of research from the aspect of node sorting, the method based on node neighbor will ignore the influence of the path between nodes, and the algorithm based on node path will not take into account the influence of the neighbor nodes. Therefore, from the perspective of interaction between nodes, Ma et al. proposed an algorithm to mine key nodes based on the universal gravity formula [17]. Based on the gravity formula, researchers have introduced multi-attribute features into the gravity formula to enhance the model’s identification precision [18]. Inspired by the spring model, Meng et al. [19] proposed the local spring model (LSM). These methods fully integrate node neighborhood and path information. Although these methods show good performance in some scenarios, they mainly focus on the local characteristics and path information of nodes, ignoring the complexity and uncertainty of the local structure of nodes.

We present a new framework in this research: the Second-order Neighborhood Betweenness Entropy Fuzzy Local Dimension Spring Model (SNEFLD-SM). Based on the spring model, this method integrates the second-order neighborhood entropy and fuzzy local dimension (FLD). The introduction of information entropy can effectively capture the local and global information of the network, and more accurately capture the location information of nodes in the local and the whole network. In order to fully consider the self-similarity and heterogeneity of the network, fuzzy local dimension is introduced to enhance the model’s differentiation of edge nodes. In large networks, it is easy to gather high-influence nodes, which leads to the “rich-club phenomenon”, resulting in ineffective information transmission. Therefore, weakening factors are introduced in this paper to alleviate this phenomenon. Most traditional key node identification methods believe that nodes have the same scope of influence, but in real networks, such as public opinion networks, communicators with high influence will be exposed to more nodes and their scope of influence will be larger than those with low influence. On this basis, this paper introduces the scope of influence of nodes and assigns the scope of influence to nodes according to the important value of each node [20], thereby improving node identification accuracy and reduces computing overhead. Six authentic datasets were utilized to validate the method’s reliability through comparison with alternative measures. The susceptible-infected (SI) epidemic model is employed to simulate the propagation process under different node identification methods. We employ Kendall’s τ statistic to assess the rank correlation between nodal importance measures and infection propagation results. Finally, the optimization effect of the FLD method on the SNEFLD-SM algorithm is demonstrated through the comparison experiment between the second-order neighborhood betweenness entropy (SNB) algorithm and the non-SNB algorithm.

This research is systematically structured as follows: the identification method is presented in the second section; the third section presents a comprehensive analysis and discussion of the experimental findings; the innovation and significance of the method are discussed in the fourth section; and the conclusion is made in the fifth section.

## 2. Materials and Methods

This section describes the framework of the SNEFLD-SM. By integrating information entropy, fractal dimension, and spring-based optimization method, the proposed model captures the local and global structural features of the network and identifies the key nodes in the complex network. We first represent relationships in the real world as complex networks, where entities are abstracted as nodes and interactions between entities are mapped as edges, thus building the structural framework of the network. In this way, we construct a graph G(V,E) based on relationships in the real world, where V represents nodes and E represents connected edges. Given this study’s emphasis on detecting crucial nodes in complex networks, we perform node screening on graph G(V,E) to remove disconnected nodes; this preprocessing step simplifies subsequent calculations of node importance and enhances computational efficiency. Secondly, we compute the second-order neighborhood centrality and betweenness centrality based on the network structure and integrate them into the information entropy framework.

**Figure 1:**
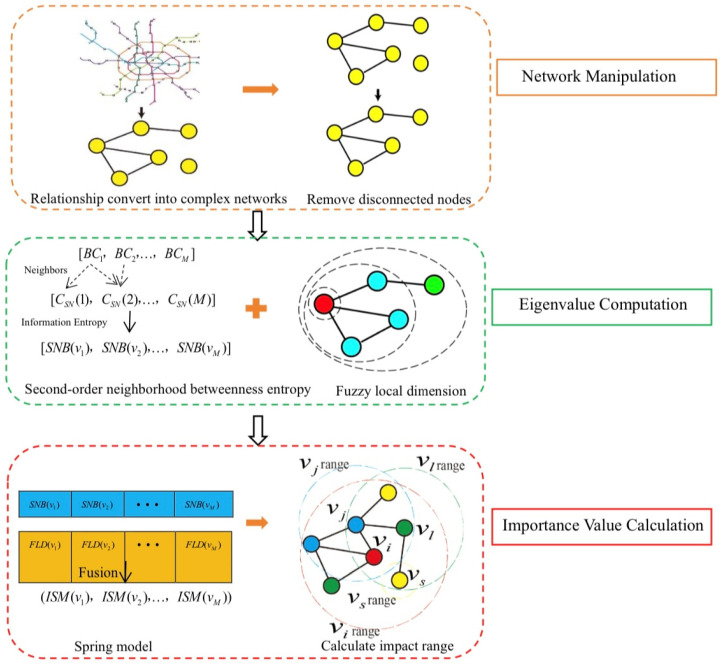
Key Node Identification Flowchart: A Topological Potential Fusion Approach.

This approach effectively captures both local and global network characteristics. To further enhance the representation of local node properties, this paper introduces the fuzzy local dimension method. This method uses the box-covering approach from fractal techniques, employing boxes of different sizes to cover the central node. Since the contributions of the covered nodes to the central node vary depending on their distances, the membership function $A_{ij}(\varepsilon )$ is used to describe the fuzzy relationship between nodes and assign different weights. Thirdly, the second-order neighborhood betweenness entropy may overlook some nodes that have significant local structural impacts but do not necessarily possess high centrality or betweenness values. In contrast, the fuzzy local dimension, due to its high sensitivity to local structural changes, is capable of identifying such nodes. Therefore, in this paper, we integrate the second-order neighborhood betweenness entropy with the fuzzy local dimension using a spring model to derive the ISM. Fourthly, the influence range of each node is calculated based on the ISM value, and a damping factor is introduced. This effectively solves the problem of information propagation caused by the aggregation of high-importance nodes. Figure 1 illustrates the computational and validation framework of the SNEFLD-SM. The models used in each step are described in detail as follows.

### 2.1. Network Manipulation

In order to mine the key nodes in the real world, we must first build a complex network according to the relationships in the real world. Where entities are abstracted as nodes, and the interactions between them are modeled as edges. The complex networks studied in this paper are unweighted and undirected networks. After the complex network framework G(V,E) is obtained, some nodes may exist independently of the largest connected subgraph. Network preprocessing ensures efficient computation and robust key node identification by filtering independent nodes and exclusively preserving the dominant connected component.

### 2.2. Eigenvalue Computation

In this section, we will elaborate on the details of generating two eigenvalues in the spring model. This section is divided into four parts. The first and second parts introduce the concepts of second-order neighborhood centrality and betweenness centrality, respectively. In the third part, information entropy is used to integrate these two centralities, obtaining the second-order neighborhood betweenness entropy. The fourth part explains the process of generating fuzzy local dimension eigenvalues based on fractal technology.

#### 2.2.1. Second-Order Neighborhood Centrality

Degree centrality is a basic measure of node centrality and a basic method to evaluate the importance of nodes in an interconnected system. This approach assumes that nodes with a higher number of connections generally exhibit greater structural impact because they are directly connected to more neighbors in the network architecture and have higher local importance. The mathematical representation of degree centrality is as follows:

(1) $$DC_{i}=\sum_{j}^{N}a_{ij}$$

Degree centrality assumes that the importance of a node in influence propagation demonstrates direct correspondence to its connection count. However, this method overemphasizes the importance of the neighbor nodes and ignores the influence of other nodes in the neighborhood of the node on its importance. In order to overcome this limitation, this work employs the second-order neighborhood centrality approach. The node centrality of this method is not only determined by its neighbors but is also affected by the node centrality in its second order neighborhood. The calculation formula is as follows:

(2) $$C_{SN}(i)=\sum_{u\in \Gamma (i)}DC_{u}$$

where Γ(i) indicates the neighbor node of node i, and $DC_{u}$ indicates the degree centrality of node u.

**Figure 2:**
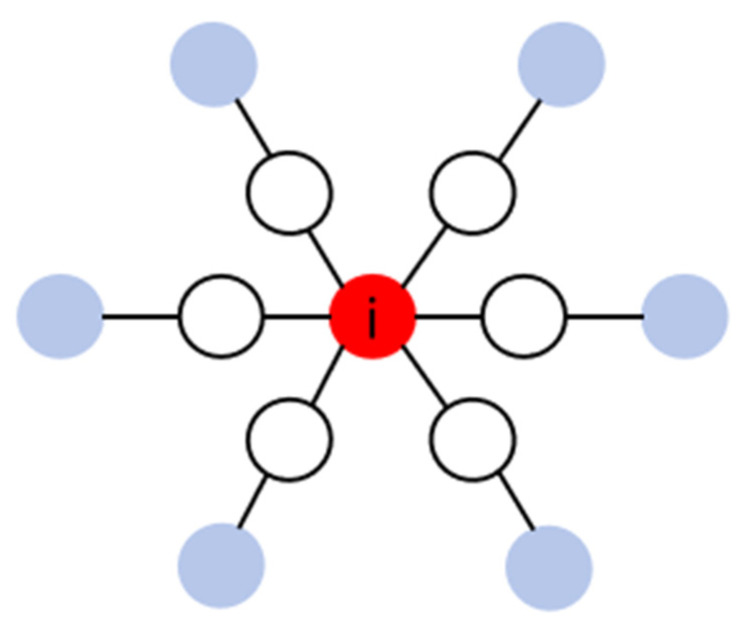
The Second-order Neighborhood Centrality (The blue nodes represent the second-order neighborhood).

To extend the analysis to the second-order neighborhood, we employ the number of nodes in the second-order range. As shown in Figure 2, compared with degree centrality (DC), this method can capture the indirect influence generated by nodes within the higher-order neighborhood, expand the analysis range of local characteristics of the network, and reflect the multi-hop nature of information propagation in the network more effectively.

#### 2.2.2. Betweenness Centrality

After capturing the local topological characteristics of nodes through second-order neighborhood centrality, the global structural attributes of nodes, such as their positional roles in the network, must also be considered to evaluate node importance from a global structural perspective. To address this, we integrate path-based information by introducing betweenness centrality (BC), a global geometric metric that quantifies the extent to which nodes function as bridging elements along optimal paths between network pairs. Formally, BC index evaluates a node’s brokerage potential by computing the ratio of the following: (a) shortest paths transiting through the target node to (b) all possible shortest paths. For node i, this is expressed as follows:

(3) $$BC(i)=\sum_{j,k\neq i}\frac{N_{jk}(i)}{N_{jk}}$$

where $N_{jk}$ is the number of shortest paths from node j to node k and $N_{jk}(i)$ is the number of shortest paths from node j to node k passing through node i. Betweenness centrality is a method used to determine whether a node is in a significant position within a graph. Specifically, when multiple shortest paths converge at a particular node, that node assumes a key bridging position in the graph structure, making it more important compared to other nodes, and, thus, it has a higher betweenness centrality.

#### 2.2.3. The Second-Order Neighborhood Betweenness Entropy

The two node centrality algorithms calculated in the previous sections have different limitations and advantages. For example, the second-order neighborhood centrality focuses on the local information in the network but ignores the global information. In contrast, betweenness centrality emphasizes global- and path-based information but fails to capture the local structure of the network. Different centrality methods will produce different node importance rankings, which is not conducive to the evaluation of node comprehensive importance. Therefore, information entropy is introduced in this paper to integrate the two centrality measures into a unified feature value to comprehensively evaluate the importance of nodes in the local and global scope of the network.

Information entropy provides a powerful framework for quantifying the uncertainty and randomness inherent in node centrality metrics within complex networks. Given that node importance varies significantly based on connection patterns, roles in information flow, and influence on network structure and dynamics, entropy-based measures offer a principled way to capture this variability. For a network G(V,E) with V nodes and E edges, the information entropy related to node importance can be defined by applying the standard entropy formula to centrality values. While Nie et al. [21] pioneered specific entropy-based local measures, their work inspires our approach. Nie et al. defined the local entropy (LE) of a node as follows:

(4) $$LE_{i}=-\sum_{j\in \Gamma (i)}DC_{j}\log DC_{j}$$

Here, $DC_{j}$ symbolizes the degree-based centrality value of node j and Γ(i) constitutes the neighborhood set of node i. Considering the node-to-neighborhood association mapping, Nie et al. defined the mapping entropy (ME) by the intersection of the degree centrality of nodes $v_{i}$ and $v_{j}$:

(5) $$ME_{i}=-DC_{i}\sum_{j\in \Gamma (i)}DC_{j}$$

where $DC_{i}$ represents the degree centrality of node i, and $DC_{j}$ represents the degree centrality of node j, which is a neighbor of node i. This method not only considers the degree of the node but also the degrees of its neighboring nodes, which helps to improve the accuracy of the algorithm.

These concepts effectively incorporate not only a node’s degree but also the degrees of its neighbors. However, LE and ME approaches, being restricted to neighborhood analysis, fail to capture nodes’ global significance. Building upon this foundation of using entropy to integrate local structural information, our work proposes a novel node importance assessment method. We leverage the core insight of entropy-based fusion but extend it significantly: (1) incorporating second-order neighborhood information to capture broader influence; and (2) integrating betweenness centrality to reflect the node’s pivotal role in information transmission paths. The second-order neighborhood betweenness entropy (SNB) is defined as follows:

(6) $$SNB(v_{i})=-BC_{i}\sum_{j\in \Gamma (i)}C_{SN}(j)$$

where $BC_{i}$ represents the betweenness centrality of node i, $C_{SN}(j)$ represents the second-order neighborhood centrality of node j, which is mentioned in Section 2.2.1, and Γ(i) represents the set of neighbor nodes of node i. Betweenness centrality considers global information of the network, while second-order neighborhood centrality reflects the local characteristics of the network. Combining these two centrality measures through information entropy can effectively improve the accuracy of critical node identification.

#### 2.2.4. Fuzzy Local Dimension and Fractal Techniques

Sliver et al. [22] considered the local properties of networks, demonstrating that power-law distributions govern not only small-world networks but also numerous empirical complex network systems and lead to the proposal of the local dimension (LD) formula. The formula indicates that the radius r is power-law distributed with the number of nodes within the radius r centered at node i. The distribution relationship is as follows:

(7) $$D_{i}=\frac{d}{d\log r}\log B_{i}(r)$$

Here, r indicates the shortest-path distance from node i; To compute the slope $D_{i}$, the metric $B_{i}(r)$ needs to be calculated for distinct values of r within the range (1,$d_{\max}$), where $d_{\max}$ represents the maximum shortest-path distance originating from node i. The function $B_{i}(r)$ enumerates nodes located within the r-hop neighborhood of i. The parameter $D_{i}$ corresponds to the slope coefficient derived from least-squares regression on log–log transformed data.

The local dimension method assigns equal consideration to all nodes located within a distance from the central node that does not exceed the specified box radius. However, in general, the distances of each node to the central node are different, and thus their contributions are also different. Nodes positioned nearer to the central node exhibit greater contribution weights in the analysis. Based on this, Wen et al. proposed the concept of fuzzy local dimension.

Fuzzy local dimension considers the local properties of nodes, and its expression is as follows:

(8) $$FLD(v_{i})=\frac{d}{d\log r_{t}}\log N_{i}(r_{t},\varepsilon )$$

where $r_{t}$ represents the distance radius to the central node i, which takes values in ascending order from the set $\left\{1,2,\ldots ,d_{i}^{\max}\right\}$, and $d_{i}^{\max}$ represents the maximum distance from node i to any other node in the network. $N_{i}(r_{t},\varepsilon )$ is the fuzzy number of nodes covered by a box of size ε obtained through fuzzy sets, defined as follows:

(9) $$N_{i}(r_{t},\varepsilon )=\frac{\sum_{j=1}^{N}A_{ij}(\varepsilon )}{N_{ir}}$$

Here, ε represents the dimension of the network box, centered around the central node, and $N_{ir}$ denotes the number of nodes covered by the box. In simpler terms, it quantifies nodes residing in the r radius neighborhood of node i. The function $A_{ij}(\varepsilon )$ maps node j to a membership value based on its containment in the box of dimension r around node i, defined by the following:

(10) $$A_{ij}(\varepsilon )=\exp(-\frac{{d_{ij}}^{2}}{\varepsilon^{2}})$$

where $d_{ij}$ denotes the minimal path length connecting node j and the central node i. The contribution of node j to the fuzzy function $N_{i}(r_{t},\varepsilon )$ of the central node i is denoted by $A_{ij}(\varepsilon )$, which has a range of [0, 1], indicating that different nodes contribute differently to the central node. If node j is closer to the central node i, its contribution to the central node increases, and $A_{ij}(\varepsilon )$ approaches 1. Conversely, if node j is farther away from the central node, its contribution to the central node decreases, and the value of $A_{ij}(\varepsilon )$ becomes smaller, approaching 0. The variation of $A_{ij}(\varepsilon )$ will be reflected in the fuzzy number $N_{i}(r_{t},\varepsilon )$ and will impact the importance of node i.

It allocates distinct significance weights to nodes according to their distances from the central node [23]. The detailed computation procedures are as follows:

1. Traverse all nodes in the network and sequentially select each node as the center node.
2. For each center node, calculate $N_{i}(r_{t},\varepsilon )$ within different radius $r_{t}$.
3. Substitute $N_{i}(r_{t},\varepsilon )$ and the corresponding radius $r_{t}$ into the double logarithmic scale fitting curve, and obtain the fuzzy local dimension $FLD(v_{i})$ based on the slope of the curve.
4. Repeat the above steps to calculate the fuzzy local dimension for all nodes in the network.

Unlike conventional centrality methods, the fuzzy local dimension (FLD) characterizes node-level attributes in complex networks through a fractal dimension perspective, focusing on local structural heterogeneity rather than global topological features. This approach assigns each node j a distance-dependent weight $A_{ij}(\varepsilon )$, where $A_{ij}(\varepsilon )$ decays with increasing shortest-path distance from the central node i. Consequently, proximate neighbors contribute more significantly to i’s importance assessment. Node criticality is then quantified by analyzing the functional relationship between the box-covering dimension and the count of nodes contained within variable-radius boxes centered at i.

### 2.3. Importance Value Calculation

#### 2.3.1. Improved Spring Model

In this section, we introduce a spring model to integrate the second-order neighborhood betweenness entropy and the fuzzy local dimension calculated in Section 2.2.3 and Section 2.2.4. The spring model simultaneously considers the proximity information and path information between nodes, incorporating the network diameter. By treating the influence between nodes as elastic forces, it effectively integrates both local and global structural features of the network. This enables the model to more accurately identify nodes that exhibit high influence from both local and global perspectives.

Meng et al. use concepts derived from Hooke’s Law to assess nodal importance [19], proposing the local spring model (LSM). Inspired by this model, this paper proposes an improved spring model (ISM) algorithm, where the importance of nodes in the network is defined as follows:

(11) $$ISM(v_{i})=\sum_{i\neq j,j\in N}L_{i}L_{j}(d-d_{ij})$$

In this formula, d represents the diameter of the network, and $d_{ij}$ denotes the shortest distance between node i and node j. The difference $d-d_{ij}$ represents the difference between the network diameter and the shortest path length between two nodes, which is considered as the deformation amount of the spring. The formula regards the influence force between two nodes as an elastic force F, treating it as a scalar that considers only its magnitude and not its direction. By summing the combined force of each node with all other nodes in the network, the importance value of each node can be obtained. In this study, we define the node value $L_{i}$ as a composite metric that quantifies node importance through dual perspectives: the second-order neighborhood betweenness entropy (SNB) and fuzzy local dimension (FLD). This methodology not only preserves critical structural characteristics of the network topology but also enhances the differentiation of peripheral nodes’ significance, thereby improving the model’s generalization capability. The formulation is presented as follows:

(12) $$L_{i}=\lambda \frac{SNB(v_{i})-SNB_{\min}}{SNB_{\max}-SNB_{\min}}+(1-\lambda )\frac{FLD(v_{i})-FLD_{\min}}{FLD_{\max}-FLD_{\min}}$$

In the formula, $SNB_{i}$ represents the second-order neighborhood betweenness entropy of node i and $FLD_{i}$ represents the fuzzy local dimension of node i. $SNB_{\min}$ and $SNB_{\max}$ denote the minimum and maximum values of the second-order neighborhood betweenness entropy for all nodes in the network, respectively. Similarly, $FLD_{\max}$ and $FLD_{\min}$ denote the minimum and maximum values of the fuzzy local dimension for all nodes in the network, respectively. λ influences coefficients that reflect the weights of SNB and FLD on the importance of the nodes. In this experiment, the parameter λ is set to 0.5, indicating that SNB and FLD are assigned equal weights in contributing to node importance values. By using this method, the second-order neighborhood betweenness entropy and the fuzzy local dimension can be effectively integrated, providing a more comprehensive consideration of the network’s structural characteristics.

#### 2.3.2. Calculate Impact Range

While Formulas (11) and (12) enable the calculation of influence for each node, this approach aggregates the interaction effects of all nodes within the network, which leads to high computational complexity and lower efficiency, potentially impacting accuracy.

The importance of each node is different. For example, in social networks, individuals with greater influence often have connections with more people in the network and can mutually affect each other. Therefore, drawing on the concept from reference [24], this study develops a spring-model-inspired mechanism to quantify nodal influence domains in complex networks. This algorithm posits that the influence range of each node varies, with more important nodes having a larger influence range, and less influential nodes having a smaller influence range.

**Figure 3:**
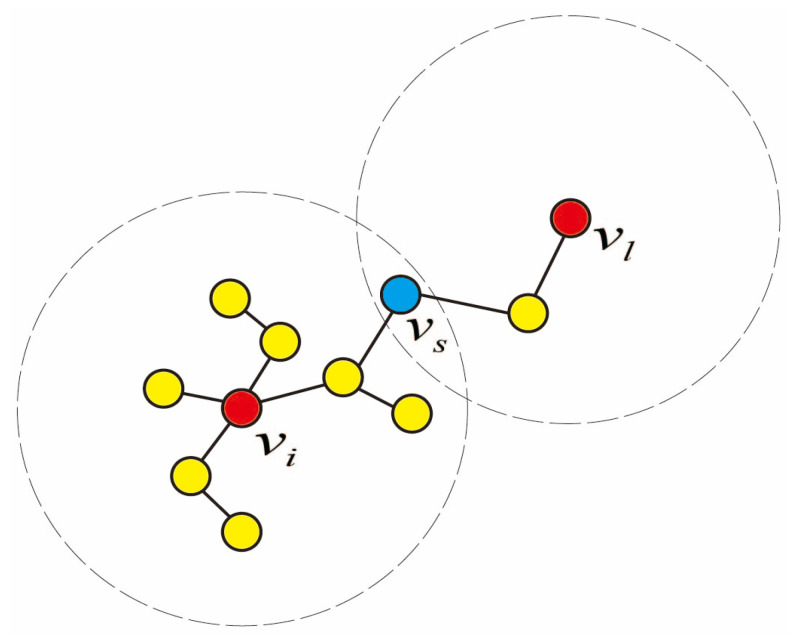
Node Influence Range.

For node $v_{i}$ in Figure 3, node $v_{l}$ is the node in the network that is the farthest from $v_{i}$. Suppose there exists a node $v_{s}$ on the path between $v_{i}$ and $v_{l}$. For node $v_{s}$, the forces exerted by nodes $v_{i}$ and $v_{l}$ on this node are equal. According to Formula (11), the force exerted by node $v_{i}$ on node $v_{s}$ is as follows:

(13) $$F_{is}=L_{i}L_{s}(d-d_{is})$$

Since the distance between node $v_{i}$ and $v_{s}$ is $d_{is}$, and the distance from node $v_{i}$ to node $v_{l}$ is $d_{il}$, the distance from node $v_{s}$ to node $v_{l}$ is $d_{il}-d_{is}$. Similarly, the influence from node $v_{l}$ can be derived as follows:

(14) $$F_{sl}=L_{s}L_{l}(d-(d_{il}-d_{is}))$$

Since $F_{is}=F_{sl}$, then the following:

(15) $$L_{i}L_{s}(d-d_{is})=L_{s}L_{l}(d-(d_{il}-d_{is}))$$

Therefore, the following:

(16) $$d_{is}=\frac{(L_{i}-L_{l})*d+L_{l}*d_{il}}{L_{l}+L_{i}}$$

In this context, $d_{is}$ represents the influence range of node $v_{i}$, and $v_{l}$ is the node farthest from $v_{i}$. Therefore, Formula (16) can be rewritten as follows:

(17) $$R_{i}=\frac{(L_{i}-L_{l})*d+L_{l}*d_{l}}{L_{l}+L_{i}}$$

Upon deriving the nodal influence boundaries through Equation (17), and combining with Formula (11), we can derive the spring model formula based on the node influence range as follows:

(18) $$ISM(v_{i})=\sum_{d_{ij}\leq R_{i}}L_{i}L_{j}(d-d_{ij})$$

Compared to the traditional SM model, the proposed ISM in this work combines SNB and FLD. SNB integrates local topological features and global path characteristics using information entropy, addressing the limitation of single methods that focus excessively on either local or global aspects. However, this approach overlooks the network’s sensitivity to local structure. Therefore, by introducing fractal techniques, ISM utilizes FLD’s high sensitivity to local structural changes. This compensates for SNB’s oversight of nodes with low betweenness but high local influence, resolving the ambiguity issue in heterogeneous structures. Finally, a spring model converts the mathematical representation into quantifiable influence.

#### 2.3.3. Attenuation Factor

In modern networks, it is common to observe a prominent clustering phenomenon where nodes with high influence tend to aggregate within specific communities, leading to the “rich club” effect. To address this issue, Guo et al. [25] proposed the EnRenew algorithm. This method involves selecting the most important nodes first and then updating the information entropy of their reachable nodes, and repeating the process.

Inspired by this approach, this paper introduces a weakening factor into the spring model. By utilizing the improved spring model to select nodes with peak influence metrics, the influence of its neighboring nodes is subsequently reduced. This approach effectively mitigates the clustering phenomenon that often occurs during information propagation. The mechanism by which the weakening factor operates is illustrated in the following formula:

(19) $$SNEFLD-SM(v_{j})=(1-\frac{ISM(v_{i})}{ISM(v_{i})+\sum_{u\in \Gamma (i)}ISM(v_{u})})*ISM(v_{j})$$

where $v_{j}\in \Gamma (i)$, Γ(i) indicates the collection of neighboring nodes for node $v_{i}$. In every iteration, the node $v_{i}$ with the most significant impact is pinpointed within the current network, subsequently diminishing the influence of its neighboring nodes. The more important a node is, the more it suppresses the importance of surrounding nodes. This mechanism facilitates efficient information dissemination within densely connected subnetworks while enhancing the overall stability of complex networks.

### 2.4. SNEFLD-SM Key Node Identification Algorithm

The SNEFLD-SM algorithm can be depicted as follows (Algorithm 1):


**Algorithm 1 Power Method**
**Input**: Network G.**Output**: Significance of each node.1. Calculate the second-order neighborhood centrality of each node according to Formula (2).2. Determine the betweenness centrality of each node using Formula (3).3. Calculate the second-order neighborhood betweenness entropy according to Formula (6).4. Calculate the fuzzy local dimension according to Formulas (7)–(10).5. Substitute the results into Formulas (11) and (12) and compute the influence range.6. **For all**
$v_{i}\in G$
**do the following**:7.    Select the node $v_{i}$ with the highest value.8.    **For all**
$v_{j}\in \Gamma (i)$
**do the following**:9.        Apply Formula (19) to reduce the value of node $v_{j}$10.  **end for**.11. **end for**.12. **return** SNEFLD-SM.13. Sequence the nodes in the list according to SNEFLD-SM.

## 3. Experimental Evaluation and Performance Analysis

### 3.1. Datasets

This experiment employed one artificial network ER and five real-world network datasets: Dolphins [26], USAir [27], Dublin Infection, Email, and Hamster. Specifically, the ER random network comprises 650 nodes with a connection probability of *p* = 0.01. The Dolphins dataset represents the social network of dolphins. The USAir dataset describes the airline network in the United States. The Dublin Infection dataset represents the contact network of infected individuals in Dublin city. The Email dataset is an email network where members can send emails to each other. Lastly, the Hamster dataset describes the social relationships among hamsters. These network datasets can be downloaded from the website https://networkrepository.com/index.php(accessed on 1 June 2025). Table 1 offers detailed information about the datasets, where $\left|N\right|$ stands for the number of nodes in the network, and $\left|V\right|$ represents the number of edges in the network. The average degree and maximum degree are represented by $\langle k\rangle$ and $k_{\max}$. The average shortest distance and maximum shortest distance are denoted by $\langle \omega \rangle$ and $\omega_{\max}$, respectively.

### 3.2. Centrality Scores of Nodes

Table 2, Table 3 and Table 4 show the ranking order (from highest to lowest) of the top ten key nodes identified by eight different methods across six datasets. JC indicates the Jaccard coefficient between each centrality method and the SNEFLD-SM method. It is clear that each method focuses on different features of the networks, causing differences in node rankings. Higher similarity in rankings between two methods suggests greater consistency in the factors they consider.

The identification results of the SNEFLD-SM method are highlighted in red, and the overlapped parts of the nodes identified by other methods and the SNEFLD-SM method are marked with colors. Nodes marked with underline have the same importance ranking as the nodes identified by SNEFLD-SM. JC denotes the Jaccard coefficient, with values ranging between 0 and 1. A JC value closer to 1 indicates higher similarity between two methods, meaning more identical nodes are identified among the top 10 important nodes. Conversely, JC = 0 signifies no overlapping nodes.

In the ER artificial network, the top ten nodes identified by the SNEFLD-SM method show high similarity with results from DC, BC, CC, BE, and HV methods, with overlap exceeding 80%. Notably, the top six nodes identified by SNEFLD-SM demonstrate identical ranking order to those identified by the BC method. This indicates that SNEFLD-SM shares similar prioritization principles with these conventional methods for node identification, demonstrating strong reliability. In the Dolphins network, the similarity between the top ten important nodes obtained by SNEFLD-SM and the results obtained by DC, BC, BE, and HV methods is as high as 60%, indicating that SNEFLD-SM and these methods have a certain consistency in the network. The identification results of SNEFLD-SM and CC method overlap 50% of the nodes. The nodes obtained by LSS and FLD methods are the most different from those obtained by SNEFLD-SM, and only 30% of the nodes are the same, indicating that SNEFLD-SM’s emphasis on mining key nodes is the most different from these methods. In the USAir network, the top 10 nodes identified by SNEFLD-SM are completely consistent with the results obtained by CC and BE methods, indicating that SNEFLD-SM, CC, and BE have similar concerns in identifying the key nodes of this network. The overlap rates of the first 10 nodes of SNEFLD-SM and DC, HV, and FLD methods are 90%, 80%, and 80%, respectively. In contrast, the overlap rate between SNEFLD-SM and the BC and LSS methods is lower, at 70%. In the Dublin Infection network, the BE method shows the highest similarity with SNEFLD-SM, with a 90% overlap in the top 10 node lists. The overlap rates between SNEFLD-SM and BC, CC, DC, FLD, and LSS are 90%, 80%, 70%, 50%, and 50%, respectively. In the email network, the top 10 nodes identified by SNEFLD-SM completely overlap with those obtained by the BC method, although their rankings differ, indicating that SNEFLD-SM and BC share the most similar considerations in this network. The overlap rates for the top 10 nodes between SNEFLD-SM and BE, CC, DC, HV, FLD, and LSS are 90%, 80%, 70%, 70%, 70%, and 70%, respectively. In the Hamster network, the BE method shows the highest similarity with SNEFLD-SM, with a 90% overlap in the top 10 nodes and nearly identical rankings. The DC, BC, CC, HV, and LSS methods differ from SNEFLD-SM by less than 30% in their top 10 node lists. However, the FLD methods show low similarity with SNEFLD-SM, with an overlap rate of less than 50%.

Analysis of the Jaccard coefficients between centrality methods and SNEFLD-SM across datasets shows that in the ER network, DC exhibited the strongest correlation 0.818 with SNEFLD-SM node rankings while showing the weakest correlation 0.111 with FLD. In the Dolphins network, DC, BC, BE, and HV shared identical Jaccard coefficients 0.429 with SNEFLD-SM. For the USAir network, both CC and BE achieved a Jaccard coefficient of 1 with SNEFLD-SM, indicating identical top 10 important nodes. In the Dublin Infection network, BE demonstrated the highest similarity with SNEFLD-SM, whereas HV showed the lowest. BC exhibited the highest similarity in the Email network, while BE achieved the maximum Jaccard coefficient 0.818 in the Hamster network.

### 3.3. Measuring Effectiveness by SI Model

This part assessed the effectiveness of the SNEFLD-SM algorithm against alternative centrality measures by applying the SI model to six open datasets. Section 3.3.1 introduces the SI model, followed by an analysis of the experimental results in Section 3.3.2.

#### 3.3.1. SI Model

The SI model is one of the most fundamental propagation models in epidemiology. It provides a mathematical framework for simulating pathogen dissemination patterns in human cohorts. Under the uniform mixing assumption, the temporal evolution of the SI epidemic model is governed by a system of ordinary differential equations. The formula is as follows:

(20) $$\frac{dS(t)}{dt}=-\frac{\beta S(t)I(t)}{N}$$

(21) $$\frac{dI(t)}{dt}=\frac{\beta S(t)I(t)}{N}$$

Here, β represents the infection rate, reflecting the efficiency of disease transmission. N represents the total population. Over time, fewer people remain susceptible, and more become infected, eventually leading to the infection of the entire population.

By simulating the disease propagation process, the SI model can effectively identify critical nodes in complex networks. These nodes typically have high connectivity or are centrally located in the network, acting as super-spreaders that significantly accelerate the spread of infection. By quantifying the influence of nodes in the propagation process, such as infection speed and the number of infections, the SI model can validate the importance of critical nodes.

#### 3.3.2. SI Infection Results Analysis

**Figure 4:**
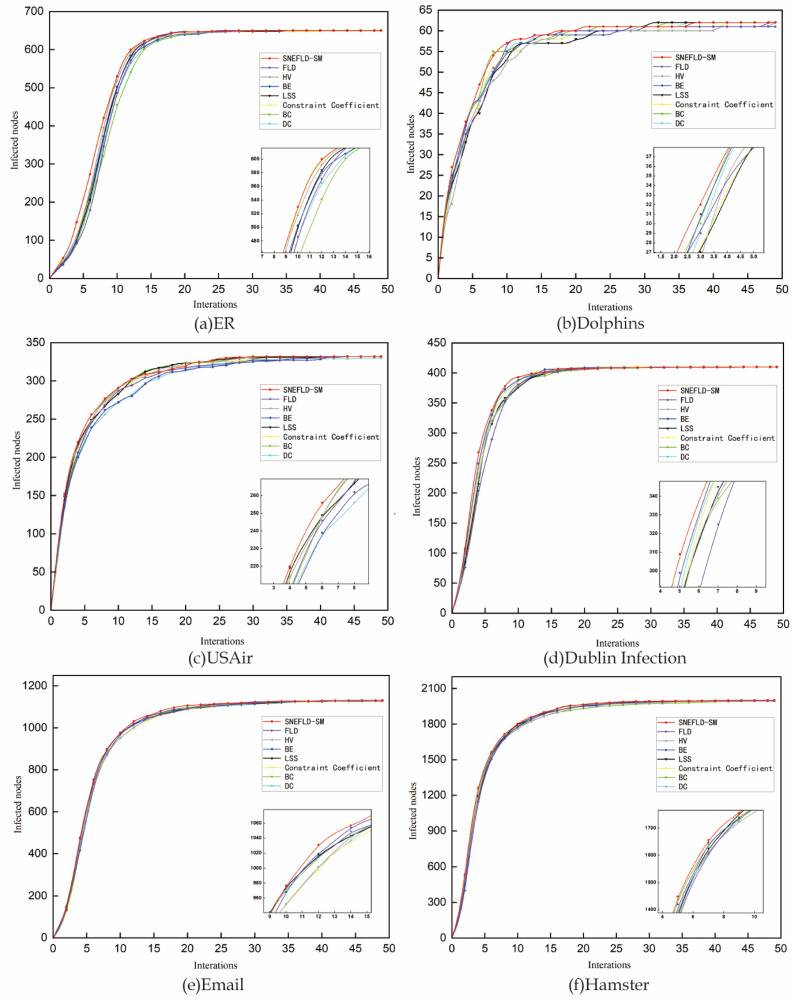
SI Infection Curves in Six Complex Networks.

The simulation seeds infection at the 10 highest-ranked nodes determined by each centrality metric in the SI framework. A higher infection rate would cause nodes to be infected too quickly, hindering the mining of critical nodes, while a lower infection rate would result in excessively slow infection speeds and prolonged infection durations. To effectively differentiate the performance of each centrality method, we set β to 0.1, avoiding the impact of excessively high or low infection rates on the results. To mitigate the randomness of the experiment, the results are averaged over 100 runs to prevent the influence of extreme values. The results as shown in Figure 4.

As shown in Figure 4a, the infection curve for SNEFLD-SM in the ER network rises faster than other methods during the early infection stage. This shows that the key nodes identified by SNEFLD-SM spread more widely and infect more nodes at the beginning of transmission. These nodes hold more important positions in the network, demonstrating the accuracy of SNEFLD-SM in ER networks. In contrast, the BC method shows the slowest curve rise, suggesting it is not well-suited for this type of network. The Dolphins network results are presented in Figure 4b. It can be observed that the curves of SNEFLD-SM, BC, DC, and BE exhibit a high degree of overlap and rise rapidly. Moreover, SNEFLD-SM demonstrates a faster propagation speed in the early stages of infection compared to other centrality methods, indicating that SNEFLD-SM is the most accurate in identifying key nodes in the Dolphins network. The curves of LSS methods rise more slowly and gradually approach the other curves as the number of iterations increase. In the USAir network, as shown in Figure 4c, it can be observed that when the iteration count is less than 10, all curves remain very close. During this period, the SNEFLD-SM curve rises the fastest and reaches the highest infection count, indicating it performs best in this network. However, after the iteration count exceeds 10, methods such as BC and DC gradually fall behind others in the growth rate of infected nodes. They also take the longest time to reach the infection peak. In the Dublin Infection, Email, and Hamster networks, the growth trends of the SI curves for the eight node centrality methods are roughly similar. The SNEFLD-SM method shows the fastest growth rate, and its infection curve is very close to those of FLD, HV, BE, LSS, CC, BC, and DC, indicating that these methods perform well in these three networks. The SNEFLD-SM method introduces a decay factor, which reduces the influence of surrounding nodes after selecting a key node, effectively addressing the impact of high-value node clustering.

It is evident that the top 10 nodes identified by SNEFLD-SM maintain a strong influence across all networks. This further validates the effectiveness and rationale behind the SNEFLD-SM method in identifying key nodes.

### 3.4. Correlations with SI Model

To assess the reliability of the SNEFLD-SM method, we employed Kendall’s tau coefficient to analyze the correlation between the importance scores derived from various centrality methods and the actual influence determined by the SI model across six real-world datasets. This experiment is structured as follows: Section 3.4.1 introduces Kendall’s tau coefficient, Section 3.4.2 defines the concept of real infection capability, and Section 3.4.3 presents the analysis of the experimental results.

#### 3.4.1. Kendall’s Tau Coefficient

Kendall’s tau coefficient is a metric used to assess the correlation between two sequences. The formula is as follows:

(22) $$\tau =\frac{C-D}{0.5n(n-1)}$$

Here, n represents the number of nodes, and C represents the number of consistent pairs, that is, the data pairs of the two variables in the same order. D represents the number of inconsistent pairs, which are the data pairs in the opposite order. According to the formula, the value of τ approaches 1 when the pairs are more concordant, while τ equals −1 if the pairs are entirely discordant.

#### 3.4.2. Real Infection Ability

In this paper, Kendall’s tau coefficient approaching unity indicates heightened concordance between nodal centrality rankings and epidemic propagation potential, which better demonstrates the reliability of the identification method. The node centrality values are calculated using the corresponding centrality methods. The actual infection capabilities are obtained using the SI infection model. When node i serves as the initial infection source, its spreading potential is quantified by the cumulative infected population size post-simulation. Elevated infection counts correlate with enhanced nodal significance.

#### 3.4.3. Kendall’s Tau Coefficient Results Analysis

**Figure 5:**
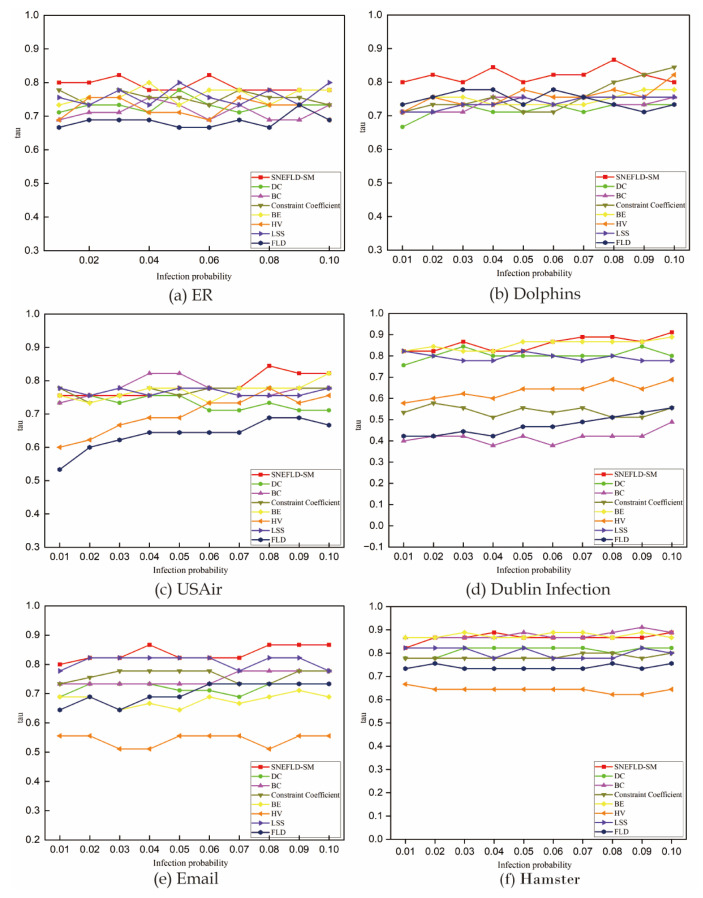
Comparison of Kendall τ values of node importance and propagation efficiency.

To mitigate the influence of excessively high or low infection rates on the actual infection capabilities of the nodes, the infection rate β in this experiment is set within the range of 0.01 to 0.1. The number of infected nodes at iteration t = 10 is used to represent the actual infection capability of each node, as it reflects the spread of infection within the network at a stable point. To minimize the impact of random fluctuations, all quantitative outcomes are stabilized through centesimal (N = 100) trial repetitions. The findings and subsequent analysis are presented as follows:

In the ER network, the τ for the centrality methods fluctuate steadily between 0.7 and 0.8. This shows a strong correlation between the infection counts and the node rankings identified by these methods, indicating that the results from the SI model are reliable. Compared to other methods, the FLD method has lower tau values when β∈(0.01,0.08). However, the tau value for FLD rises notably when β is greater than 0.08. The results for the Dolphins network are shown in Figure 5b. The τ of SNEFLD-SM fluctuates slightly within the range of β∈(0.01,0.1), with the overall tau value ranging between 0.8 and 0.85, which is higher than the other six centrality methods. The CC curve rises significantly as the tau value increases. The tau values of the remaining centrality methods remain stable. For the USAir network, the τ of SNEFLD-SM shows an upward trend in the ranges of 0.04 < β < 0.05 and 0.07 < β < 0.08, reaching its peak at = 0.08, and then slightly decreasing when β > 0.08. Meanwhile, the curves for both HV and FLD increase gradually as β rises. In the Dublin Infection network, the τ of SNEFLD-SM decreases when 0.03 < β < 0.05, performing worse than BE. It rises when 0.05 < β < 0.07 and outperforms BE when β > 0.06. In the Email network, SNEFLD-SM and LSS outperform the other six centrality methods. The τ of SNEFLD-SM is at its minimum of 0.8 when β = 0.01, reaches its maximum of 0.86 when β = 0.08, and then remains stable thereafter. In the Hamster network, all centrality methods remain stable. The performance of SNEFLD-SM is slightly inferior to that of BE and BC methods, with its τ consistently around 0.87.

### 3.5. Assessing the Impact of the Second-Order Neighborhood Betweenness Entropy on SNEFLD-SM Algorithm

**Figure 6:**
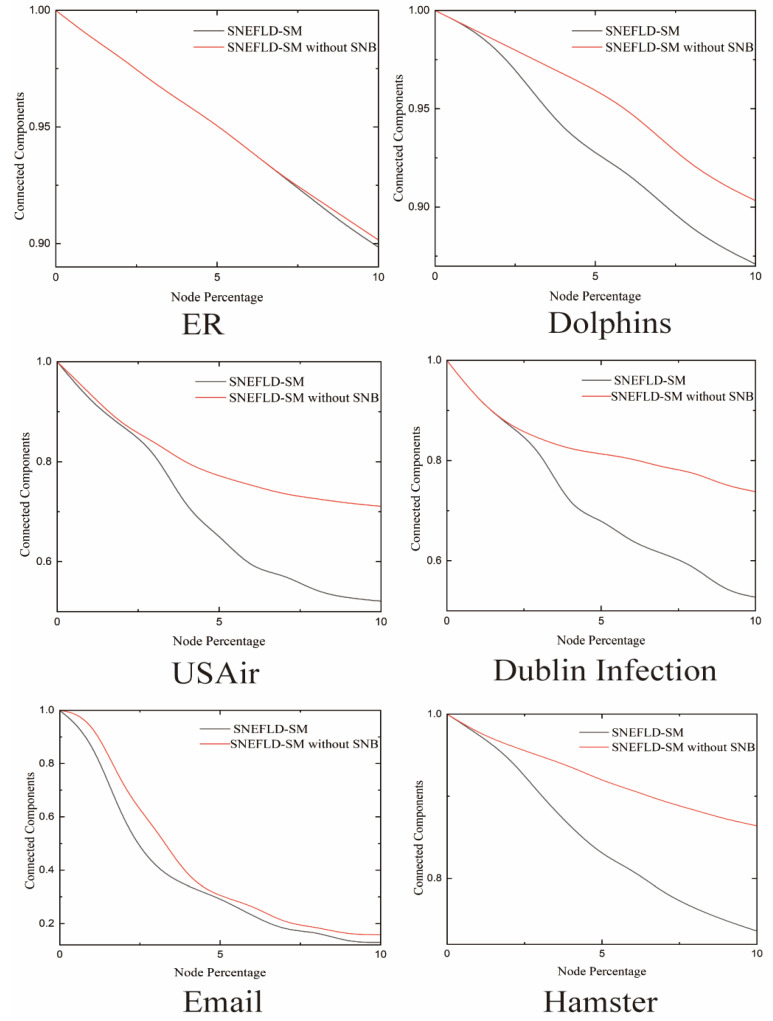
Comparative Analysis of Connected Components: SNEFLD-SM Model vs. SNEFLD-SM without SNB Model.

To further test how well the SNB algorithm captures both local and global information in complex networks and gives a more complete picture of key nodes, we set up a comparison experiment using real-world networks [29]. We use the SNEFLD-SM model and its variant without SNB (called SNEFLD-SM without SNB) to rank node importance in six typical complex networks. Then, we removed nodes one by one starting from the most important ones [30]. We watched how the network connectivity changed during this process by plotting how the connected components (CC) change as we remove more nodes (shown in Figure 6).

In the ER network, the performance curves of the standard SNEFLD-SM and SNEFLD-SM without SNB algorithms coincide when removing the top 6% of nodes. This indicates that the SNB component contributes minimally to identifying the most critical 6% of nodes. However, when node removal increases to the 6–10% range, the network connectivity components decline more rapidly under the standard SNEFLD-SM method. This demonstrates that nodes identified using the SNB algorithm possess greater importance in network fragmentation. In the Dolphins network, as the percentage of nodes removed increases, the SNEFLD-SM without SNB algorithm keeps the connectivity at 0.95 when removing the first 5% of key nodes. Meanwhile, the standard SNEFLD-SM algorithm drops to 0.93. When 10% of nodes are removed, SNEFLD-SM without SNB still keeps the core connectivity above 0.90, while the SNEFLD-SM algorithm goes down to around 0.85. Both curves show a declining trend, and the SNEFLD-SM without SNB curve stays above the SNEFLD-SM curve the whole time. The difference between them grows bigger as more nodes are removed, but later stops growing. This shows that when the SNB algorithm is added, the nodes found by the SNEFLD-SM algorithm become more important. Removing them causes more damage to the network. Therefore, the SNEFLD-SM algorithm works better in this network. In the USAir, Dublin Infection, and Hamster networks, the change trends for the algorithms with and without SNB are similar. Networks using the SNEFLD-SM algorithm still have fewer connected components than networks using SNEFLD-SM without SNB. And the gap in connected components becomes bigger as more nodes are removed. This indicates that after incorporating the SNB algorithm, the critical nodes identified by the SNEFLD-SM algorithm play more critical roles in maintaining the overall integrity of the network. Also, as attacks remove more parts of the network, the advantage of the SNB algorithm becomes more obvious. In the Email network, as nodes are removed step by step, both methods show decreasing connectivity. But with SNB added to SNEFLD-SM, the network breaks down much faster. When 3% of nodes are removed, the standard SNEFLD-SM connectivity drops sharply below 0.4, while SNEFLD-SM without SNB stays above 0.5 at this stage. However, between 4% and 10% node removal, the difference in connectivity between the two methods becomes smaller.

Overall, the SNEFLD-SM method works better than SNEFLD-SM without SNB at finding key nodes in all six networks. When using SNEFLD-SM, the connected components of the network break down faster. This shows that the important nodes found by SNEFLD-SM cause more damage to the network when removed, meaning these nodes are more critical.

### 3.6. Time Complexity

The computational efficiency of centrality algorithms is key to their application in large-scale complex networks. Here, we compare traditional methods referenced in the study and analyze the time complexity of the proposed SNEFLD-SM framework. DC has a linear time complexity of O(n), where n is the number of nodes. Betweenness centrality (BC) relies on global shortest paths, resulting in O(nm) complexity for unweighted networks, where m is the number of edges. CC similarly has O(n^3^) complexity. The LSS algorithm involves local structure scanning and neighborhood aggregation, leading to O(n^2^) complexity in networks.

The SNEFLD-SM algorithm combines SNB and FLD, but its influence range calculation restricts computations to a dynamically set radius, reducing its complexity to O(n⟨r⟩), where ⟨r⟩ is the average influence range. As a result, SNEFLD-SM delivers better node identification accuracy with lower computational cost.

## 4. Discussion

This study employs undirected and unweighted networks based on the following considerations: First, such networks represent the most fundamental and thoroughly investigated models in network science. The theoretical foundations and standard implementations of numerous classical propagation models including the SI model and centrality metrics are established upon these networks. This provides our research with a clear and reproducible benchmark analysis framework, enabling direct and unambiguous comparison of results with a vast body of the existing literature, thereby facilitating fundamental-level validation of our model’s core mechanisms. Second, in undirected unweighted networks, differences in propagation paths and node influence originate primarily from the network topology itself, such as degree distribution, clustering coefficient, and connectivity, rather than from heterogeneity in link attributes like weight, direction, and sign. This enables a more isolated examination and clearer understanding of the impact of novel mechanisms introduced by our model on the propagation process, while avoiding interference from multiple confounding factors.

The SNEFLD-SM model proposed in this study addresses the inherent limitations of traditional node centrality methods in complex networks. Conventional approaches, such as degree centrality (DC) and betweenness centrality (BC), predominantly focus on either local or global network structures without effectively integrating both perspectives. While advanced methods like PageRank and semi-local centrality (SLC) attempt to bridge this gap, they still fall short in capturing the intricate interplay between local neighborhood influences and global network topology [31]. This limitation becomes particularly pronounced in dynamic and heterogeneous networks, where a node’s significance is determined not only by its direct neighbors but also by its position within the entire network.

To overcome these challenges, SNEFLD-SM integrates multiple centrality measures, including second-order neighborhood centrality, betweenness centrality, and fuzzy local dimension (FLD), within a spring model framework. By introducing information entropy, the model effectively integrates the local and global structural features of complex networks, offering a more comprehensive evaluation of node importance. Moreover, the introduction of FLD, grounded in fractal theory, enhances the model’s ability to handle the diversity and uncertainty of nodes in highly heterogeneous networks. This enhancement significantly improves the model’s generalization capability, making it applicable across various complex network scenarios. Furthermore, in networks characterized by high-impact node clustering, which often impedes efficient information propagation, SNEFLD-SM introduces a weakening factor to mitigate this effect, facilitating a more effective diffusion process.

Experimental results strongly validate the effectiveness of SNEFLD-SM across multiple datasets. Comparative analyses with traditional centrality methods reveal that key nodes identified by SNEFLD-SM exhibit superior performance in the SI model, demonstrating higher accuracy in capturing influential nodes. The Kendall’s tau coefficient analysis further confirms a strong correlation between SNEFLD-SM-ranked key nodes and their actual infection capabilities, underscoring the robustness of the proposed approach. Additionally, comparative experiments highlight that the integration of the SNB module into the SNEFLD-SM framework significantly enhances its ability to detect critical nodes, particularly in maintaining network connectivity. These findings not only validate the effectiveness of SNB in refining node importance evaluation but also demonstrate its optimization effect on the SNEFLD-SM algorithm. Experiments illustrate SNEFLD-SM’s practical efficacy on complex networks, contributing positively to future developments in the field.

Regarding future research directions, generalizing the model’s core mechanisms to weighted and directed networks holds significant value. For instance, applying this model to international trade networks would enable the modeling of policy shock propagation dynamics [32]. Analysis of trade network structures can effectively elucidate the systemic implications of trade policies or tariff changes on interstate alliances and trade agreements.

## 5. Conclusions

This study introduces the SNEFLD-SM model, which integrates multiple centrality measures, such as second-order neighborhood centrality, betweenness centrality, and fuzzy local dimension, within a spring model framework. The model effectively addresses the limitations of traditional methods and enhances the accuracy of key node identification. The SNEFLD-SM framework shows advantages over traditional methods in multiple networks, with the identification of key nodes exhibiting stronger correlation with actual propagation capabilities. Moreover, the incorporation of the SNB module significantly optimizes the model’s node importance evaluation, providing a valuable foundation for future research.

## Figures and Tables

**Table 1:** The topological properties of six networks.

Network	$\left|N\right|$	$\left|V\right|$	$\langle k\rangle$	$k_{\max}$	$\langle \omega \rangle$	$\omega_{\max}$
ER	650	2138	7	17	3.643	7
Dolphins	62	159	5	12	3.303	8
USAir	332	2126	12	139	2.730	6
Dublin Infection	410	2765	13	50	3.622	9
Email	1133	5451	9	71	3.603	8
Hamster	2423	16630	13	273	3.586	10

**Table 2:** Top decile nodes identified by eight centrality measures in ER and Dolphins networks.

Rank	ER								Dolphins							
	SNEFLD-SM	DC	BC	CC	LSS	BE	HV	FLD	SNEFLD-SM	DC	BC	CC	LSS	BE	HV	FLD
1	35	35	35	35	35	35	35	511	38	15	37	37	37	37	15	38
2	393	394	393	393	393	393	394	61	18	38	2	38	21	38	46	21
3	394	393	394	394	394	394	393	35	21	46	41	21	30	2	38	15
4	468	286	468	286	351	468	286	126	58	52	38	15	2	41	34	37
5	511	465	511	351	465	286	465	164	41	34	8	52	15	21	21	41
6	286	482	286	428	598	598	482	621	52	18	18	2	51	18	58	34
7	465	425	425	465	511	425	351	568	14	58	21	30	41	15	14	51
8	425	351	598	511	468	465	598	213	48	21	55	18	38	55	30	46
9	103	511	465	103	10	482	425	391	54	30	52	41	44	52	41	44
10	351	468	634	468	428	511	468	582	50	41	58	44	25	58	52	19
JC	\	0.818	0.667	0.818	0.539	0.667	0.667	0.111	\	0.429	0.429	0.333	0.177	0.429	0.429	0.177

**Table 3:** Top decile nodes identified by eight centrality measures in USAir and Dublin Infection networks.

Rank	USAir								Dublin Infection							
	SNEFLD-SM	DC	BC	CC	LSS [28]	BE	HV	FLD	SNEFLD-SM	DC	BC	CC	LSS	BE	HV	FLD
1	118	118	118	118	118	118	118	118	157	157	274	157	148	157	148	274
2	261	261	8	261	261	261	261	261	304	304	304	304	157	304	372	157
3	182	255	261	182	255	182	255	67	274	148	157	274	274	274	157	333
4	255	152	201	255	152	255	182	255	243	372	243	333	205	243	304	1
5	152	182	47	201	182	201	166	201	333	282	333	148	223	333	286	243
6	201	230	182	152	230	152	152	182	148	217	30	372	304	148	60	150
7	67	166	255	67	166	47	230	166	372	314	212	243	193	372	318	305
8	230	67	152	166	112	67	67	47	30	333	148	305	239	30	116	205
9	47	112	313	230	67	230	112	248	212	286	297	1	282	282	291	314
10	166	201	13	47	147	166	147	112	314	60	218	217	243	314	217	337
JC	\	0.818	0.539	1.000	0.667	1.000	0.667	0.667	\	0.429	0.667	0.539	0.333	0.818	0.250	0.333

**Table 4:** Top decile nodes identified by eight centrality measures in Email and Hamster networks.

Rank	Email								Hamster							
	SNEFLD-SM	DC	BC	CC	LSS	BE	HV	FLD	SNEFLD-SM	DC	BC	CC	LSS	BE	HV	FLD
1	105	105	333	23	233	105	105	333	73	73	73	73	73	73	73	69
2	333	333	105	333	135	333	333	23	121	121	6	69	121	121	121	131
3	23	16	23	105	578	23	23	42	69	301	69	121	301	69	301	73
4	42	23	578	135	23	42	16	105	6	202	121	202	189	6	202	136
5	41	42	76	233	333	76	42	76	301	6	13	240	202	301	69	66
6	233	41	233	578	76	41	41	468	202	69	301	6	313	202	6	622
7	76	196	135	41	52	233	233	41	66	189	66	301	6	66	313	617
8	578	233	41	42	105	578	196	233	13	313	21	66	69	13	622	612
9	135	21	355	378	332	135	76	52	21	622	159	242	84	21	617	121
10	355	76	42	52	183	16	354	378	189	617	2	13	622	622	189	13
JC	\	0.539	1.000	0.667	0.539	0.818	0.539	0.539	\	0.539	0.666	0.666	0.539	0.818	0.539	0.333

## Data Availability

The data presented in this study are available on request from the corresponding author.
